# Vertigo in Pregnancy: A Narrative Review

**DOI:** 10.7759/cureus.25386

**Published:** 2022-05-27

**Authors:** Luis Carlos Serna-Hoyos, Andres Felipe Herrón Arango, Santiago Ortiz-Mesa, Sara Maria Vieira-Rios, Daniela Arbelaez-Lelion, Johanna Marcela Vanegas-Munera, Melissa Castillo-Bustamante

**Affiliations:** 1 Gynecology, Clinica Universitaria Bolivariana, Universidad Pontificia Bolivariana, Medellín, COL; 2 Otolaryngology - Head and Neck Surgery, Medical School, Universidad Pontificia Bolivariana, Medellín, COL; 3 Gynecology, Medical School, Universidad Pontificia Bolivariana, Medellín, COL; 4 Epidemiology and Public Health, Medical School, Universidad Pontificia Bolivariana, Medellín, COL; 5 Medical School, Health Sciences School, Universidad Pontificia Bolivariana, Medellín, COL; 6 Otolaryngology - Head and Neck Surgery, Massachusetts Eye and Ear Infirmary, Boston, USA

**Keywords:** vestibulocochlear nerve diseases, vestibular disorders, pregnancy, dizziness, vertigo diagnosis

## Abstract

During pregnancy, physical, hormonal, and psychological changes may occur from conception to labor. Balance is also impacted throughout this time, leading to symptoms such as vertigo and unsteadiness. These symptoms may appear at any time and can cause disability and physical impairment. Little has been published about vertigo in pregnancy. We performed a narrative review of vertigo in pregnant patients. Vertigo in pregnant females may be associated with hormonal changes in peripheral structures and inner ear organs. Meniere’s disease, vestibular migraine, and benign paroxysmal positional vertigo are usually exacerbated during pregnancy. Specific changes to hearing and proprioception in the physical examination are also noted between the second and third trimester of pregnancy. These symptoms are usually seen in pregnant patients throughout this time. Some types of vertigo may be exacerbated and others may present at any time of pregnancy. Further research is needed to understand the clinical and pathological association of audiovestibular symptoms during pregnancy.

## Introduction and background

Pregnancy is a nine-month-long physiologic process with psychological, cardiovascular, and hormonal changes that affect several organs. Many of these changes are modulated by hormones, including estrogen, progesterone, human chorionic gonadotropin, placental lactogen, and relaxin, leading to anatomic and functional changes in the respiratory, cardiovascular, gastrointestinal, musculoskeletal, cutaneous, and audio-vestibular systems [1].

Several conditions affecting the audio-vestibular system, such as hearing loss, otosclerosis, tinnitus, autophony, facial paralysis, and vertigo, present for the first time or get exacerbated during pregnancy [2]. This can be explained by the action of estrogen and progesterone on specific structures involved in balance and hearing, such as the cochlea, stria vascularis, and spiral ligament, leading to chemical and osmolar changes in the endolymphatic fluid, which is a critical substance involved in the regulation of inner ear functions [3]. However, these are not the only structures affected by the influx of hormones [4]. Proprioception and cognition may also be altered due to electrolytic and vascular action on peripheral receptors leading to gait disturbances and falls [5].

To date, few data have revealed that vertigo is exacerbated in patients with a previous history of Meniere’s disease during pregnancy and some others have reported their onset to be associated with vestibular neuritis; however, there is a lack of information regarding this symptom throughout pregnancy [2]. Another challenge during this time is the presentation of syncope, unsteadiness, and dysautonomia, which are usually reported by pregnant women, making the diagnosis and follow-up of vertigo even more difficult for clinicians and otolaryngologists. The aim of this study was to discuss the prevalence, clinical manifestations, and types of vertigo usually seen in pregnant patients.

Methodology

This literature search was conducted in PubMed, Scopus, EBSCO, Bvsalud, and Scielo to search for articles published between January 2000 and December 2021 in English and Spanish languages using the following medical subject heading (MeSH) terms: “dizziness,” “vertigo,” “pregnancy,” “vestibular disease,” and “vestibulocochlear nerve diseases” and boolean operators AND/OR. The inclusion criteria were articles reporting vertigo during pregnancy including the following types of studies: case reports, retrospective chart reviews, cross-sectional studies, case-control cohorts, and systematic reviews. Additionally, articles reporting Meniere’s disease, vestibular neuritis, benign paroxysmal positional vertigo (BPPV), and other presentations of peripheral vertigo during pregnancy were included. Editorials, narrative reviews, scope reviews, letters to the editors, comments, and abstracts were excluded. Articles not focused on vertigo during pregnancy and those focused on vertigo before pregnancy or during adolescence were also excluded. Reports of vertigo in pregnant females associated with inner ear malformations, arteriovenous malformations, and central nervous diseases were also excluded. In total, 73 articles were found, of which 10 articles were included in this review. All articles selected were cross-checked by the authors.

## Review

Vertigo in pregnancy: pathology and clinical events

Vertigo is defined as the false sensation of movement [6-8]. This perception can be a rotating perception of the environment (objective) or of oneself (subjective) [8]. At least 80% of the global population has suffered one episode of vertigo over their lifetime, and it is considered one of the most common complaints in emergency rooms and office consultations [8,9]. Globally, the annual incidence is around 7% and rises up to 30% per year [9]. Females are usually affected (1:2.7) compared to males worldwide [9]. This is a disabling and incapacitating symptom associated with altered function in the inner ear in up to 70% of cases [9]. However, disturbances to the function and anatomy of the inner ear are not the only factors associated with the onset of vertigo [8]. Proprioception, vision, and central nervous integration are also implicated in the balance and pathogenesis of vertigo [8]. As several connecting pathways are also implicated near vestibular systems such as the protuberance, flocculus, spinal cord, and vomiting center, other symptoms may appear concomitant to vertigo, such as vomiting, nausea, unsteadiness, gait imbalance, and falls [9-13].

Vertigo is one of the most common complaints reported by pregnant patients to primary care physicians [14]. In the United States, 32 pregnant females for every 100,000 new cases per year visit a primary care physician complaining of vertigo [14,15]. As a common complaint, primary care physicians, gynecologists, and otolaryngologists usually manage these patients; however, it is rarely studied as it is widely common and associated with metabolic, orthostatic, and functional changes [15].

It can be potentially explained by genetic predisposition, hormonal fluctuation, and metabolic changes throughout a patient’s lifetime [11,12]. During the fertile decades, mainly in pregnancy, this symptom may be frequent but remains underdiagnosed. Pregnancy is the period between the fertilization of an egg by a sperm and birth lasting nine months, during which time estrogens and steroidal sex hormones are increasingly produced, making several changes to the functioning of diverse organs, such as the adrenal gland and placenta [11,12]. However, others such as the vestibular system, integrated by the inner ear, visual system, proprioception, and central nervous system may also be affected [13].

The inner ear is responsible for two crucial functions, namely, hearing and balance. Hormonal changes in the endolymphatic fluid may alter the homeostasis and enzymatic processes at the cochlea, stria vascularis, spiral ligament, and spiral ganglion neurons, as well as at the afferent terminations of neurons. Moreover, estrogen affects some enzymatic receptors at the sodium-potassium channels throughout the cochlea and membranous labyrinth leading to the presentation of otologic symptoms in pregnant patients [16-19]. Other events described in relation to the inner ear include the fluid retention in endolymph and perilymph and hypercoagulability in the auditory arteries.

The labyrinth vascularization is caused by the labyrinthine artery and its branches [16-19]. These are highly vulnerable to vascular occlusion [16-19]. In addition to the probable occlusion, mostly seen during the second month of pregnancy and stabilized in the second or third trimester [19], an increase in some coagulation factors (VII, VIII, IX, X, XII) and fibrinogen are noted, along with a decrease in factor XI [19]. Therefore, pregnancy represents a state of hypercoagulability with greater activation of the blood coagulation and fibrinolysis system, which can increase plasma viscosity and erythrocyte aggregation and decrease deformability. Red blood cells in pregnant women cause an increased risk of thromboembolism in the labyrinthine artery and vascular occlusion in the labyrinthine microcirculation [19].

During pregnancy, the production and excretion rates of sex steroid hormones (estrogen and progesterone) are significantly increased [19]. In the third trimester, circulating progesterone levels are 20 times higher than basal levels and estradiol levels are 30 to 40 times higher than during the normal menstrual cycle [20]. These hormonal changes may cause electrolytic imbalance due to excessive retention of sodium and water, which leads to an increase in the volume of extracellular fluid [20].

Other changes in estrogen during pregnancy are associated with the role of brain function wherein estrogen levels are associated with difficulties in spatial orientation during the various gestational weeks [7]. Hormonal variations during pregnancy can, in turn, cause a sensation of aural fullness and hearing loss at low frequencies, with a decrease in hearing levels at frequencies of 125, 250, and 500 Hz in the first trimester and a subsequent increase in the second and third trimester, which can resolve after delivery [16]. Naftalin et al. have described remission of symptoms of neurological disorders such as aural fullness and vertigo presenting in early pregnancy, with exacerbation of symptoms during the second trimester and lactation.

Vertigo (22.72%) is more frequent during the first trimester; in the second trimester, instability (12.12%) and gait imbalance (12.12%) are more frequent [10]. In the third trimester, instability (14.81%) is more frequent, followed by a tendency to fall (11.11%) [10]. This suggests that the vestibular alteration derived from the hormonal alteration causes the symptoms of vertigo in the first trimester, and this symptom in the following trimesters would occur due to labyrinthine habituation [10]. There was a reduction in balance in pregnant women in the second and third trimesters compared to non-pregnant women. In addition to these symptoms persisting into the postpartum period, there was no correlation between balance and weight gain, suggesting that postural instability in this population is more associated with hormonal, ligament, and joint changes than with abdominal enlargement or weight gain.

Most frequent types of vertigo during pregnancy

Several types of vertigo are seen during pregnancy. Some of them are exacerbated in patients with previous diagnoses of vestibular disorders, such as Meniere’s disease or vestibular migraine [5]. Exacerbations of Meniere’s disease are mostly seen during the third trimester of pregnancy in up to 57% of patients and vestibular migraine in up to 50% of patients [5]. Other events common during pregnancy are BPPV. These common pathologies are described below.

Meniere’s Disease

Meniere’s disease is an episodic type of vertigo, associated with disturbances in the regulation of endolymphatic fluid in the inner ear, leading to obstruction, increased endolymphatic flow, and volumetric changes that may be represented clinically by hearing loss, tinnitus, fullness, and vertigo [21]. In addition to these characteristics, several environmental, genetic, and metabolic causes are associated [21]. During pregnancy, there is a reduction in the osmolality of systemic and local fluids to the ear, leading to an increased and turbulent osmotic gradient called hydrops to the endolymphatic sac, saccule, cochlea, and semicircular canals [7].

In pregnant patients, Meniere’s disease is usually attributed to the sudden onset of hearing loss or vertigo during the second and third trimesters. Many patients first present with Meniere’s disease during pregnancy which may be reversed after labor. In patients with a previous diagnosis of Meniere’s, it may be exacerbated during the second and third trimesters [18-21]. Some patients are controlled during the first trimester and show improvement in symptoms [21]. Meniere’s disease diagnosis is confirmed using the Barany Society Criteria: (1) two or more episodes of spontaneous vertigo lasting between 20 minutes and 12 hours. (2) Low and mid-frequency sensorineural hearing loss documented with audiometry in one ear, defining the affected ear on at least one episode before, during, or after one of the vertigo attacks. (3) Fluctuating auditory symptoms (hearing loss, tinnitus, or fullness) in the affected ear. (4) There is no other vestibular diagnosis that better explains the symptoms [21].

Regarding the treatment of Meniere’s disease during pregnancy, salt and caffeine reduction are recommended [18]. Betahistine is not usually prescribed during pregnancy but in some cases must be given with caution [18]. The use of prochlorperazine in acute episodes of vertigo should also be used with caution because antipsychotics are associated with extrapyramidal effects in the newborn when used in the third trimester [18].

Benign Paroxysmal Positional Vertigo

BPPV is defined as an episodic type of vertigo triggered by positional changes and movement rotations [22]. This is the most common peripheral vestibular pathology and is observed more frequently in women than in men with a ratio of 2:1 [22]. Involvement of the posterior semicircular canal occurs more frequently compared to the lateral semicircular canal, also known as the horizontal canal [22]. Posterior canal BPPV accounts for approximately 85% to 95% of BPPV cases [22].

BPPV is associated with the presence of otoconia that migrates from the utricle and may abnormally adhere to the cupula, causing cupulolithiasis. These dense particles freely moving through the endolymphatic fluid at the semicircular canals may cause abnormal stimulation of the vestibular system, leading to canalolithiasis [22].

During pregnancy, BPPV may be exacerbated due to prolonged bed rest, sleeping on the left side (as it is recommended to reduce compression of the vena cava during pregnancy), calcium metabolism disorders, and vitamin D deficiency during the second trimester associated with higher metabolic demands of the fetus and increased resorption of calcium in several systems such as bone and kidneys [23].

Vestibular Migraine

Vestibular migraine is characterized as episodic vertigo associated with diverse symptoms such as phonophobia, light intolerance, migraine auras, and headache [24,25]. Most patients are previously diagnosed with migraine [24]. Vestibular migraine can occur at any age, and it has a prevalence of 1.1-3.2%. It is more common in women compared to men, with a ratio of 1.5-5 [24]. Although the cause of vestibular migraine remains unknown [24], various theories have been proposed, including genetic, neurochemical, and inflammatory mechanisms, all of which are derived from the pathophysiology of migraine [24].

Up to 40% of pregnant patients will experience vestibular migraine, and the duration of vertigo may vary in these patients from minutes to hours. In these patients, besides the common symptoms related to vestibular migraine, patients may frequently complain of osmophobia and tinnitus in both ears compared to non-pregnant patients [24].

Persistent positional nystagmus or saccadic pursuits are usually seen in patients with long-standing vestibular migraine in videonystagmography [25]. Other findings include reduced unilateral caloric responses, increased contralateral preponderance, and increased vestibular unilateral deficits in 10-20% of pregnant patients [25].

## Conclusions

Pregnant patients may present with vertigo, and several types of this symptom may indicate diverse vestibular disorders such as vestibular migraine, BPPV, and Meniere’s disease. Vascular and hormonal changes may be involved in the pathogenesis of vertigo from the onset of pregnancy to labor. Further clinical studies are needed to understand how vertigo affects each trimester, and how it can potentially affect fetal development.

## References

[1] Pascual ZN, Langaker MD (2021). Physiology, pregnancy. StatPearls Publishing.

[2] Pérez Rodríguez AF, Roche M, Larrañaga C (2009). [Medical disorders and pregnancy. Gastrointestinal, neurological, cardiovascular and dermatological disorders]. An Sist Sanit Navar.

[3] Shiny Sherlie V, Varghese A (2014). ENT changes of pregnancy and its management. Indian J Otolaryngol Head Neck Surg.

[4] Pechenkova E, Nosikova I, Rumshiskaya A (2019). Alterations of functional brain connectivity after long-duration spaceflight as revealed by fMRI. Front Physiol.

[5] Castillo-Bustamante M, Del Cid Chua C, Vázquez M, Bello Dotel L, Baez Recalde M (2020). Hormonas sexuales y alteraciones neurotológicas en Mujeres. Rev Fac Cien Med Univ Nac Cordoba.

[6] Çoban K, Yiğit N, Aydın E (2017). Benign paroxysmal positional vertigo in pregnancy. Turk Arch Otorhinolaryngol.

[7] Uchide K, Suzuki N, Takiguchi T, Terada S, Inoue M (1997). The possible effect of pregnancy on Ménière's disease. ORL J Otorhinolaryngol Relat Spec.

[8] Young P, Castillo-Bustamante M, Almirón CJ, Bruetman JE, Finn BC, Ricardo MA, Binetti AC (2018). [Approach to patients with vertigo]. Medicina (B Aires).

[9] Neuhauser HK, Lempert T (2009). Vertigo: epidemiologic aspects. Semin Neurol.

[10] Swain SK, Pati BK, Mohanty JN (2020). Otological manifestations in pregnant women - a study at a tertiary care hospital of eastern India. J Otol.

[11] National Cancer Institute (2021). National Cancer Institute. Pregnancy. https://seer.cancer.gov/statfacts/html/leuks.html.

[12] Baki H (2013). Estrogen and growth hormone and their roles in reproductive function. Int J Animal Vet Adv.

[13] Hansen L, Sobol SM, Abelson TI (1986). Otolaryngologic manifestations of pregnancy. J Fam Pract.

[14] Black FO (2002). Maternal susceptibility to nausea and vomiting of pregnancy: is the vestibular system involved?. Am J Obstet Gynecol.

[15] Sloane PD (1989). Dizziness in primary care. Results from the National Ambulatory Medical Care Survey. J Fam Pract.

[16] Sennaroglu G, Belgin E (2001). Audiological findings in pregnancy. J Laryngol Otol.

[17] Naftalin L, Mallett KJ (1980). Case report of ?hormonal vertigo. J Laryngol Otol.

[18] Kumar R, Hayhurst KL, Robson AK (2011). Ear, nose, and throat manifestations during pregnancy. Otolaryngol Head Neck Surg.

[19] Xie S, Wu X (2020). Clinical management and progress in sudden sensorineural hearing loss during pregnancy. J Int Med Res.

[20] Wu PH, Cheng PW, Young YH (2017). Inner ear disorders in 68 pregnant women: a 20-year experience. Clin Otolaryngol.

[21] Lopez-Escamez JA, Carey J, Chung WH (2016). [Diagnostic criteria for Menière's disease. Consensus document of the Bárány Society, the Japan Society for Equilibrium Research, the European Academy of Otology and Neurotology (EAONO), the American Academy of Otolaryngology-Head and Neck Surgery (AAO-HNS) and the Korean Balance Society]. Acta Otorrinolaringol Esp.

[22] Bhattacharyya N, Gubbels SP, Schwartz SR (2017). Clinical practice guideline: benign paroxysmal positional vertigo (update). Otolaryngol Head Neck Surg.

[23] Giacomini PG, Napolitano B, Alessandrini M, Di Girolamo S, Magrini A (2006). Recurrent paroxysmal positional vertigo related to oral contraceptive treatment. Gynecol Endocrinol.

[24] Lempert T, Neuhauser H (2009). Epidemiology of vertigo, migraine and vestibular migraine. J Neurol.

[25] Goldbrunner R, Weller M, Regis J (2020). EANO guideline on the diagnosis and treatment of vestibular schwannoma. Neuro Oncol.

